# Supplementary material for: MetaRibo-Seq measures translation in microbiomes
Source: Nat Commun. 2020 Jun 29;11:3268. doi: 10.1038/s41467-020-17081-z (PMC7324362; doi:10.1038/s41467-020-17081-z)
Supplement: Supplementary file 10 — Supplementary Data 7 [file 41467_2020_17081_MOESM10_ESM.zip › File2/Confidence_VeryHigh_Taxonomy/276511_out.krona.html]

Javascript must be enabled to view this page.

members
magnitude
magnitudeUnassigned
count
unassigned
taxon
rank

276511\_out

5

5
2
superkingdom

5
976
phylum

5
200643
class

order
171549
5

4
2005525
family

genus
4
375288


SRS012969\_contig\_number\_contig-100\_632.233834SRS023526\_contig\_number\_30371SRS077849\_contig\_number\_contig-100\_24875.107677SRS077849\_contig\_number\_contig-100\_2317.182908
species
823
4

1
815
family

816
1
genus


SRS013800\_contig\_number\_23235
species
371601
1
